# Circulating mitochondrial biomarkers in acute coronary syndrome

**DOI:** 10.3389/fmed.2025.1568305

**Published:** 2025-05-15

**Authors:** Andrea Iboleon-Jimenez, María J. Sánchez-Quintero, Ada D. M. Carmona-Segovia, Bélen Sojo, Ana María Fernández-Ramos, Luis García-Rodríguez, Ana I. Molina-Ramos, José Manuel García-Pinilla, Manuel Jimenez-Navarro, Almudena Ortega-Gomez

**Affiliations:** ^1^Unidad Docente Multiprofesional de Atención Familiar y Comunitaria, Distrito de Atención Primaria Málaga-Guadalhorce; Faculty of Medicine, University of Málaga, Málaga, Spain; ^2^Instituto de Investigación Biomédica de Málaga y Plataforma en Nanomedicina (IBIMA Plataforma BIONAND), Málaga, Spain; ^3^Department of Cardiology and Cardiovascular Surgery, Virgen de la Victoria University Hospital, Málaga, Spain; ^4^Centro de Investigación Biomédica en Red de Enfermedades Cardiovasculares (CIBERCV), Instituto de Salud Carlos III, Madrid, Spain; ^5^Endocrinology and Nutrition UGC, Victoria Virgen University Hospital, Málaga, Spain; ^6^Clinical Analysis UGC, Hospital Universitario Virgen de la Victoria, Málaga, Spain; ^7^Department of Dermatology and Medicine, Faculty of Medicine, University of Malaga, Málaga, Spain; ^8^Centro de Investigación Biomédica en Red de la Fisiopatología de la Obesidad y Nutrición (CIBEROBN), Instituto de Salud Carlos III, Madrid, Spain

**Keywords:** acute coronary syndrome, cardiovascular diseases, mitochondrial biomarkers, sex, sex dimorphism

## Abstract

**Background:**

Acute coronary syndrome (ACS) is the leading cause of mortality in developed countries. Mitochondrial dysfunction is a hallmark of various cardiometabolic diseases, including ACS. Emerging evidence suggests that evaluating mitochondrial biomarkers in plasma may offer valuable insights into the pathophysiology and management of these conditions. The present study aims to analyse the effect of ACS, sex and their interaction on plasma levels of mitochondrial markers, such as peroxisome proliferator-activated receptor gamma coactivator 1-alpha (PGC-1α), mitochondrial open reading frame of the 12S rRNA type-c (MOTS-c) and citrate syntetase (CS).

**Methods:**

A total of 18 ACS patients (8 women and 10 men) and 20 controls (8 women and 12 men) were included in this study. Venous blood samples were collected from participants after a 12-h overnight fast. Plasma levels of mitochondrial PGC-1α, MOTS-c and CS were measured.

**Results:**

ACS significantly reduced plasma levels of PGC-1α and MOTS-c. Sex did not shown a significant effect on these markers. Additionally, MOTS-c positively correlated with the first troponin and hemoglobin, PGC-1α negatively correlated with glucose and positively with HDL-cholesterol, and CS showed negative correlations with NT-proBNP, C-reactive protein, and hemoglobin.

**Conclusion:**

Mitochondria markers, MOTS-c and PGC-1α, are altered in ACS patients, with no observed sex differences. These findings represent an initial step toward integrating personalized medicine into the clinical management of ACS. Nonetheless, further studies are required to fully elucidate the role of these markers in this pathology.

## Introduction

Cardiovascular diseases (CVD) stand as the foremost contributor to global mortality, responsible for approximately 17.9 million deaths annually, alongside a substantial burden of disability-adjusted life years (DALYs) across the globe (1). Within this spectrum, acute coronary syndrome (ACS) emerges as the primary cause of mortality in developed countries. ACS is distinguished by acute or chronic ischemia resulting from an inadequate supply of myocardial oxygen and refers to a group of conditions that include ST-elevation myocardial infarction, non-ST elevation myocardial infarction, and unstable angina (2). Although morbidity and mortality rates for ACS have decreased compared to data from previous decades due to advancements in medical treatments, protocols, and the implementation of clinical practice guidelines (3, 4), the incidence rate continues to rise (5). Notably, the prevalence of ACS exhibits a gender disparity, with a higher incidence among men (639,469 individuals) compared to women (452,364) (6). This discrepancy accounts for an estimated 23 and 18% of all CVD deaths, respectively (6). However, as it has been reported, CVD in women continues to be understudied, insufficiently recognized, frequently underdiagnosed, and undertreated (7).

The clinical spectrum of ACS is exceedingly broad, with diverse debut presentations that span from cardiac arrest, electrical or hemodynamic instability with cardiogenic shock, to cases where chest pain has already subsided upon arrival at the hospital (8). Therefore, the early diagnosis and management of ACS are crucial if we aim to prevent a fatal outcome in these patients. Unfortunately, the risk period for these individuals does not conclude with their hospital discharge, as this population remains susceptible to high probabilities of readmission for any cause (3).

The pathobiology of ACS has been studied in the last years, with coronary atherosclerosis being the primary cause of ischemic heart disease (9, 10). ACS episodes are triggered by acute changes in coronary plaques, especially vulnerable thin-cap fibroatheromas, leading to endothelial dysfunction, platelet aggregation, and plaque rupture (11, 12). However, mitigating myocardial damage and reducing ACS morbidity and mortality remains challenging, highlighting the need for more effective treatments (13). A deeper understanding of myocardial ischemic injury is driving new strategies to limit myocardial damage, prevent remodeling, and preserve viable cardiac tissue. Thus, further research into the pathophysiology of ACS is essential to develop tailored and more effective therapies.

Current biomarkers for monitoring the development of ACS primarily consist of troponin I (TnI) and troponin T (TnT) (14). However, the measurement of these molecules often yields false-positive results, as their plasma levels can be elevated in various conditions, such as heart failure, sepsis, chronic kidney disease. Additionally, cardiac troponin levels in the blood only increase after myocardial cell death, which typically occurs 2–4 h post-ischemic event, and persisting detectably for days. Consequently, there is a critical need to identify new strategies for the early detection and monitoring of ACS, potentially reducing mortality.

The underlying mechanism of ACS involves reduced blood and oxygen flow to the heart muscle usually due to atherosclerosis of the coronary arteries. Mitochondrial dysfunction contributes to both the initiation and progression of atherosclerosis, making it a potential target for ACS treatment (15). Mitochondria are essential for energy production in cardiac cells, and their malfunction leads to excessive oxidant generation, which damages cellular components (15). Several studies have reported mitochondrial alterations in ACS patients, suggesting that mitochondria may be useful for detection, monitoring, or treatment of ACS (14, 15). Further research is needed to fully explore their role. Therefore, the present study aims to evaluate serum mitochondrial biomarkers in patients with ACS, while also assessing the influence of sex to these findings.

## Materials and methods

### Patient selection

A total of 18 ACS patients (8 women and 10 men) were included in the study after being evaluated at Hospital Universitario Virgen de la Victoria, Málaga (Spain). The control group consisted of 20 sex-matched healthy individuals (8 women and 12 men). Clinical evaluation included blood tests, electrocardiogram, echocardiogram, and invasive coronary angiography, and body mass index (BMI) calculation for each subject. All patients presented severe stenosis and/or regurgitation, and all of them were indicated for aortic valve replacement. The inclusion criteria were as follows: (1) patients with significant aortic valve disease, stenosis and/or insufficiency, presenting clinical indications for surgical intervention; (2) age greater than 65 years; (3) absence of mitochondrial pathology.

The study was approved by the Ethics and Research Committee of the Provincial Research of Málaga (Spain), and all participants gave their written informed consent.

### Samples collection and biochemical determinations

Levels of troponins, NT-proBNP and reactive C protein were measured at the time of admission to the hospital’s emergency room. Peripheral venous blood samples were collected from patients after a 12-h fast the morning following their admission. Control subjects also provided fasting blood samples after a 12-h overnight fast. Serum and plasma was separated into aliquots and immediately frozen at −80°C until their analysis. Serum glucose, cholesterol, high-density lipoprotein (HDL), low-density lipoprotein (LDL), triglycerides, urea, creatinine and glomerular filtration were determined by standard enzymatic methods.

### Circulating mitochondrial markers

To explore the role of mitochondria in ACS, we selected two key proteins involved in mitochondrial activity, PGC-1α and mitochondrial open reading frame of the 12S rRNA type-c (MOTS-c), analyzed their plasma levels in the studied population. Additionally, we measured citrate synthase (CS) levels as a marker of mitochondrial mass. Quantitative measurement of PGC-1α, MOTS-c, and CS were performed in plasma from both patients and controls using ELISA kits from MyBioSource (San Diego, CA, United States). Specifically, the human PGC-1α, MOTS-c, and CS ELISA kits were used, following the manufacturer’s instructions.

### Statistical analysis

The results are expressed as either mean values ± standard deviation (SD) or standard error of the mean (SEM). Clinical and metabolic data as well as mitochondrial markers were submitted to univariate general linear model with Bonferroni *post-hoc* comparison. After testing the normal distribution of the continuous variables by the Shapiro–Wilk test, we applied logarithmic transformation as needed to ensure normality of skewed variables in general linear model analysis. Group (controls and ACS patients) and sex (men and women) was considered between-subjects effects and age as a covariable. Differences by sex in cardiac parameters were evaluated using the *t*-test for independent samples or the Mann–Whitney *U* test, depending on the normality of the data distribution. Spearman correlation tests were run to assess correlation between variables. A *p*-value lower than 0.05 was considered statistically significant.

## Results

The clinical data of all selected patients and control subjects are summarized in Tables 1, 2. The results indicate statistical differences in cholesterol levels (both total, HDL, and LDL) and age between controls and ACS patients. The significantly lower LDL cholesterol levels in ACS patients may be attributed to the more intensive medical care and treatments typically received by these patients. Creatinine levels were elevated in men. Moreover, no interaction between sex and group was observed. In the sex-stratified analysis shown in Table 2, no significant differences were observed in the various troponin measurements or NT-proBNP values. However, a trend was observed, with men generally exhibiting higher levels of C-reactive protein (CRP).

**Table 1:** Characteristics of the studied subjects.

	Controls		ACS patients		*p*-value		
	Women	Men	Women	Men	Sex	Group	Interaction
*N*	8	12	8	10			
BMI (kg/m^2^)	25 ± 3	27 ± 3	25 ± 2	26 ± 2	0.199	0.915	0.469
Age (years)	69 ± 7	69 ± 5	72 ± 3	73 ± 2	0.835	0.022	0.727
Glucose (mg/dL)	98 ± 11	105 ± 8	124 ± 33	137 ± 79	0.489	0.053	0.867
Trygliceride (mg/dL)	124 ± 53	127 ± 48	118 ± 39	122 ± 53	0.830	0.707	0.978
Cholesterol (mg/dL)	227 ± 25	200 ± 18	162 ± 39	159 ± 33	0.144	<0.001	0.225
HDL-chol (mg/dL)	67 ± 18	52 ± 11	41 ± 6	34 ± 4	0.013	<0.001	0.312
LDL-chol (mg/dL)	135 ± 23	123 ± 20	94 ± 35	98 ± 26	0.651	0.001	0.379
Creatinine (mg/dL)	0.82 ± 0.14	1.03 ± 0.23	0.92 ± 0.27	1.09 ± 0.32	0.30	0.719	0.752
Urea (mg/dL)	40 ± 11	43 ± 9	45 ± 17	45 ± 17	0.779	0.702	0.763

ACS, acute coronary syndrome; BMI, body mass index; HDL-chol, high density lipoprotein-cholesterol; LDL-chol, low density lipoprotein-cholesterol. Data are mean ± SD.

**Table 2:** Relevant parameters in acute coronary syndrome patients.

	ACS patients		*p*-value
	Women	Men	Sex
*N*	8	10	
First troponin (ng/L)	8,097 ± 7,005	1,234 ± 431	1.000
Second troponin (ng/L)	21,293 ± 14,844	19,906 ± 14,204	0.401
Maximum troponin (ng/L)	21,338 ± 14,835	15,017 ± 11,584	0.722
NT-proBNP (pg/mL)	1,655 ± 441	2,430 ± 878	0.412
C-reactive protein (mg/L)	7 ± 1	26 ± 8	0.062
Hemoglobin (g/dL)	13 ± 2	14 ± 2	0.137

ACS, acute coronary syndrome; NT-proBNP, N-terminal pro B-type natriuretic peptide. Data are mean ± SEM.

**Figure 1:**
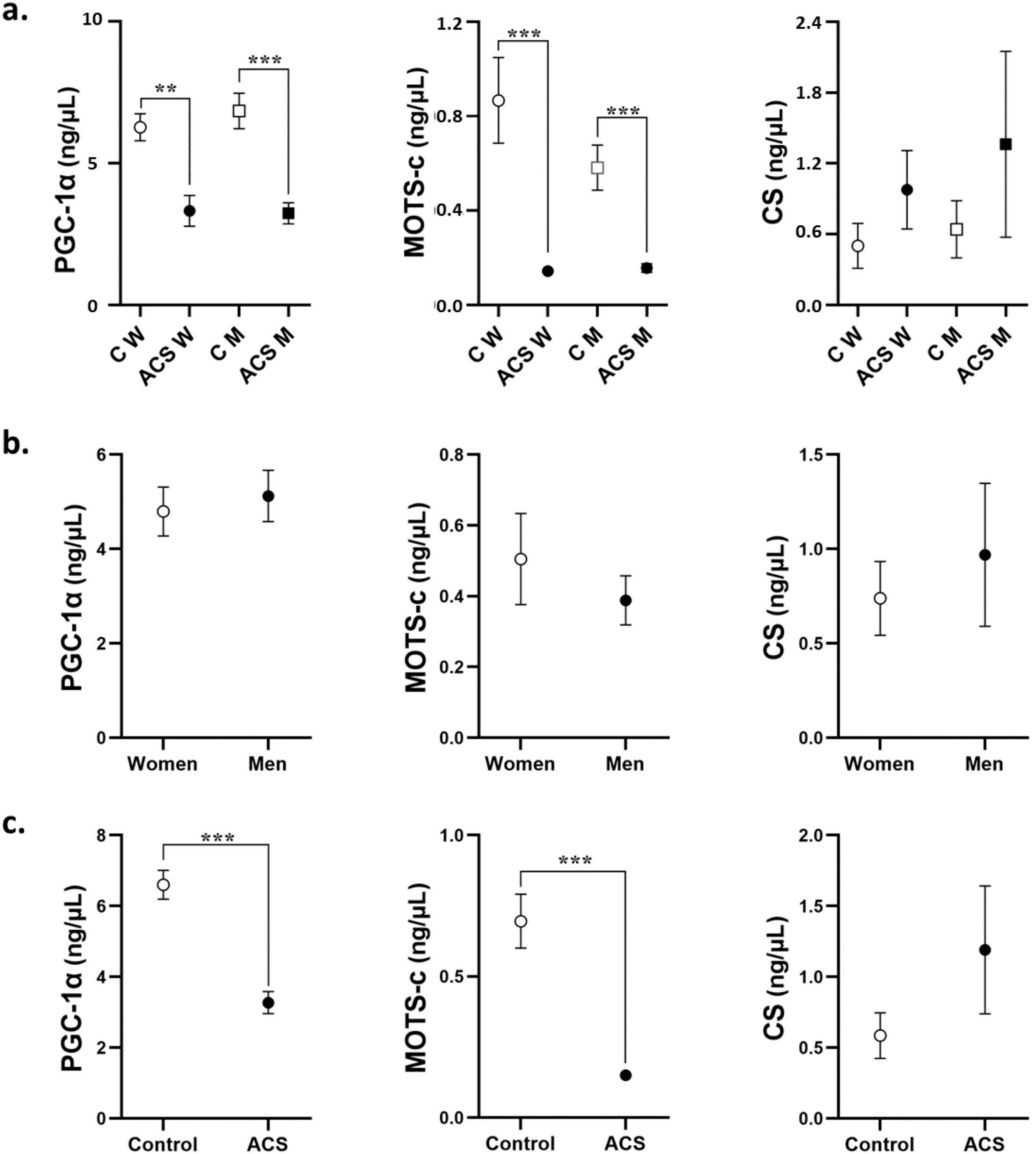
Levels of PGC-1α, MOTS-c, and CS in women and men controls and ACS patients **(a)**. The effect of sex **(b)** and ACS **(c)** on the levels of mitochondrial markers are shown. Univariate general linear model with Bonferroni *post-hoc* comparison. Data are mean ± SEM. ^**^p < 0.01 and ^***^p < 0.001. ACS, acute coronary syndrome; C, controls; M, men; W, women.

When stratifying subjects by sex and pathology (Figure 1a), we observed that both PGC-1α and MOTS-c levels were significantly decreased in the ACS group compared to the control group, with the reduction in MOTS-c levels being particularly pronounced in women (*p* < 0.0001). No significant differences were observed in circulating mitochondrial biomarker levels between men and women (Figure 1b), although MOTS-c levels tended to be higher in women. Figure 1c illustrates circulating mitochondrial biomarkers (PGC-1α, MOTS-c, and CS) in the control and ACS groups, highlighting the effect of the pathology. While PGC-1α and MOTS-c levels were significantly reduced in individuals with ACS, CS levels did not significantly differ between the two groups.

**Figure 2:**
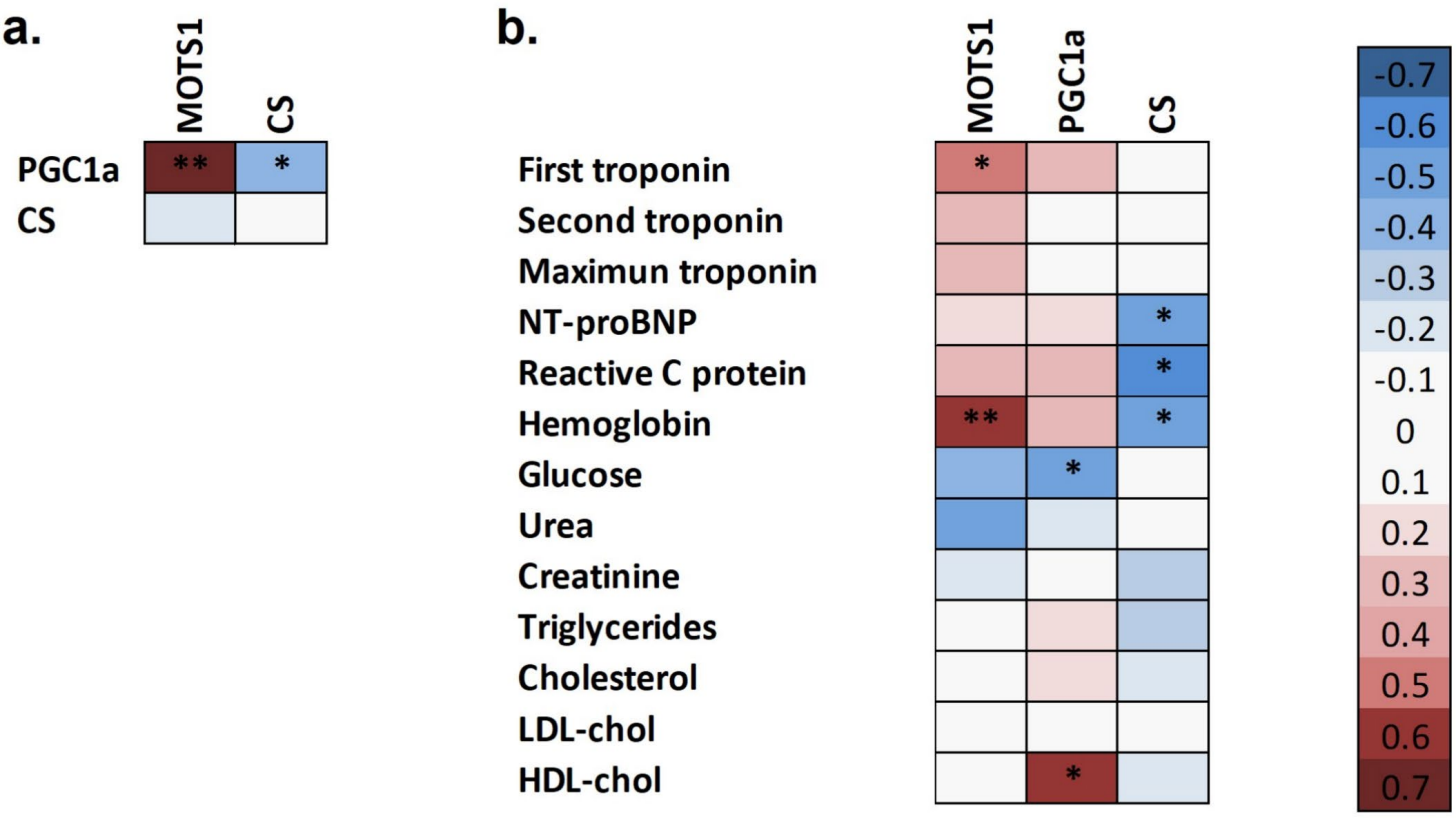
Spearman correlation analysis between mitochondrial markers in the total subjects of the study **(a)**. Spearman correlation analysis between mitochondrial markers and biochemical parameters in ACS patients **(b)**.

The correlation analysis between mitochondrial biomarkers are depicted in Figure 2a. PGC-1α shows a negative correlation with CS and a strong positive correlation with MOTS-c. Figure 2b shows correlations between mitochondrial biomarkers and cardiac and metabolic biochemical parameters. The results reveal positive correlations between MOTS-c and cardiac biochemical parameters with statistically significant correlations observed for the first troponin value, and a strong significant positive relationship with hemoglobin. PGC-1α negatively correlated with glucose and positively with HDL-cholesterol, while CS showed negative correlations with NT-proBNP, C-reactive protein, and hemoglobin.

## Discussion

We found that PGC-1α and MOTS-c levels were decreased in the serum of ACS patients compared to controls, while CS levels were increased though the differences were not significant. These findings suggest that mitochondrial function are altered in patients with ACS. Additionally, no significant differences in these mitochondrial markers were observed between men and women.

Mitochondrial metabolism is increasingly recognized as a crucial area of study for understanding the pathophysiological mechanisms involved in cardiac remodeling in ACS. Cardiomyocytes, specialized for continuous contractile activity, have very high energy demands that are met by their abundant mitochondria. These organelles play a crucial role in the cardiomyocyte response to stress, acting as a signaling hub for changes in cellular energetics, redox balance, contractile function, and cell death. Cardiac remodeling involves alterations in mitochondrial form and function, which can be either compensatory, to maintain contractility, or maladaptive (16). Mitochondrial dysfunction exacerbates ischemic damage by limiting the heart’s ability to cope with reduced oxygen supply (17), leading to impaired ATP production and increased oxidative stress due to dysregulation of Ca^2+^ uptake dynamics (18), both of which contribute to the weakening of cardiac muscle.

As the global incidence of ACS continues to rise, there is a clear need for new methodologies in diagnosing and monitoring this condition. Consequently, mitochondrial biomarkers such as PGC-1α and MOTS-c hold promise for the diagnosis, monitoring, and targeted treatment of ACS. The utility of cardiac mitochondrial biomarkers has previously been explored in other heart conditions, such as familial dilated cardiomyopathy (DCM) and familial hypertrophic cardiomyopathy (HCM) (19). Both conditions have been characterized by impaired mitochondrial enzymatic activity, highlighting the critical role of mitochondrial dysfunction in cardiac pathophysiology. We have demonstrated that MOTS-c levels are reduced in ACS. MOTS-c, a mitochondria-derived peptide, has been studied in various contexts as a potential mitochondrial biomarker (20–22). Furthermore, MOTS-c has been shown to significantly reduce pro-inflammatory factors and play a protective role against cardiac dysfunction and pathological remodeling (23). In line with our results, Yaşar et al. (24) also reported also lower MOTS-c levels in ACS patients, although their control group consisted of non-healthy individuals. A preclinical study in mice (20) have further highlighted the potential of MOTS-c as a protective agent against cardiac fibrosis under conditions of pressure overload. Administration of MOTS-c was associated with reduced levels of fibrosis and apoptosis in cardiac tissue. This metabolic protective function allows MOTS-c to modulate crucial metabolic processes in cardiac tissue during acute insults, such as those occurring in ACS, where cardiac tissue viability is severely compromised. Consistent with this, Chen et al. (25) demonstrated that ACS patients exhibited increased oxidative stress. Moreover, our study show a positive correlation between MOST-c and hemoglobin levels in ACS. The concomitant presence of hypoxic conditions, such as anemia, further compromises myocardial viability due to an increase in metabolic demand and cellular stress (26). Interestingly, correlation analysis within ACS subjects revealed a positive association between MOTS-c levels and the first troponin measurement, suggesting that during the initial hours of ACS, cardiomyocytes may attempt to counteract the acute damage. These findings emphasize the potential importance of MOTS-c in CVD. Therefore, further studies exploring the role of MOTS-c in CVD, particularly in ACS, could contribute to open new therapeutic approaches to a condition critical to cardiovascular health.

In addition, PGC-1α levels were also decreased in ACS patients in our study and showed a positive correlation with favorable metabolic status, as indicated by lower glucose levels and higher HDL-cholesterol levels. PGC-1α is a metabolic sensor that enables the body to respond to a plethora of stimuli, including exercise, fasting, and fluctuations in metabolic substrate availability (27). Our results may indicate a compromised ability to respond effectively to metabolic demands, which could contribute to the progression of cardiovascular conditions. Mitochondrial dysfunction is not only implicated in the pathophysiology of cardiovascular diseases but also plays a key role in various neurodegenerative disorders, such as Parkinson or Alzheimer disease, as well as in acute kidney injury (28). Given the systemic impact of mitochondrial function, it is crucial to consider how comorbidities present in the study population may influence the obtained results, potentially affecting the interpretation of findings and their clinical applicability. In line with our results, Li et al. (29) have showed that cardiac PGC-1α protein abundance was decreased after ischemia/reperfusion injury in a mouse model. Moreover, a PGC-1α deficient mouse model was associated with cardiac dysfunction (30). These findings contribute to the growing body of research suggesting that PGC-1α could serve as a potential biomarker for heart damage, although it does not currently replace classical ACS markers. The present study highlights the crucial role of mitochondria in ACS pathophysiology, suggesting that these markers may complement traditional biomarkers and could even serve as potential prognostic or diagnostic tools for the management of ACS patients. However, further research is needed to elucidate the underlying role of PGC-1α in ACS.

Regarding sex, our study does not show significant differences between sex and mitochondrial biomarker levels. This result could indicate that this mitochondrial biomarkers are not influenced by sex. Despite historically being dominated by men (31), CVD, particularly ACS, are also increasingly prevalent in women. This trend is partly attributed to the evolving social roles of women and increased exposure to stress, psychosocial factors, and lifestyle changes. Recent studies have emphasized that these factors contribute to a higher risk of ACS in women, especially in younger populations (32).

Although our study did not observe sex-based differences, previous research has reported disparities in the prevalence, clinical manifestations and underlying causes of CVD between men and women (33). Our study has certain limitations that should be acknowledged. The main limitation is the reduced sample size, which may affect the statistical power and the generalizability of our findings. In addition, all patients included in our cohort presented an extreme phenotype of ACS, characterized by severe stenosis and/or regurgitation, and were all indicated for aortic valve replacement. As a result, our conclusions are specific to this particular subset of patients and may not be extrapolated to the broader ACS population. Future studies including a broader spectrum of ACS patients will be necessary to confirm and expand our findings. Therefore, further studies should be performed to confirm our results, and our findings should be interpreted with caution, considering these limitations. On the other hand, our main strength lies in incorporating sex-based analysis, which reduces variability and highlights potential biological differences. Incorporating biological sex as a criterion in clinical research could further advance the development of personalized medicine.

In conclusion, mitochondria markers, MOTS-c and PGC-1α, are altered in ACS patients, with no observed sex differences. These findings represent an initial step toward integrating personalized medicine into the clinical management of ACS. Nonetheless, further studies are required to fully elucidate the role of these markers in this pathology.

## Data Availability

The original contributions presented in the study are included in the article/supplementary material, further inquiries can be directed to the corresponding author.
